# Untargeted Metabolomics Reveals Key Differences Between Yak, Buffalo, and Cow Colostrum Based on UHPLC-ESI-MS/MS

**DOI:** 10.3390/foods14020232

**Published:** 2025-01-13

**Authors:** Yuzhuo Wang, Changhui Li, Jiaxiang Huang, Qingkun Zeng, Ling Li, Pan Yang, Pengjie Wang, Min Chu, Jie Luo, Fazheng Ren, Hao Zhang

**Affiliations:** 1 College of Food Science and Nutritional Engineering, China Agricultural University, Beijing 100083, China; wyz@cau.edu.cn (Y.W.); s20223061206@cau.edu.cn (C.L.); 2 Guangxi Buffalo Research Institute, Chinese Academy of Agricultural Sciences, Nanning 530001, China; huangjx080@163.com (J.H.); zqk456@163.com (Q.Z.); lling2010@163.com (L.L.); 18434172125@163.com (P.Y.); 3 Beijing Laboratory of Food Quality and Safety, Department of Nutrition and Health, China Agricultural University, Beijing 100091, China; wpj1019@cau.edu.cn (P.W.); renfazheng@cau.edu.cn (F.R.); 4 Lanzhou Institute of Husbandry and Pharmaceutical Sciences, Chinese Academy of Agricultural Science, Lanzhou 730050, China; chumin@caas.cn; 5 College of Food Science and Technology, Hunan Agricultural University, Changsha 410114, China; luojie@hunau.edu.cn; 6 Food Laboratory of Zhongyuan, Luohe 462300, China

**Keywords:** untargeted metabolomics, yak colostrum, buffalo colostrum, cow colostrum, specific milk composition, colostrum biomarkers

## Abstract

Background: Colostrum, abundant in immunoglobulins and growth factors, plays a vital role in supporting immunity. Both yak and buffalo milk are characterized by their high protein and fat content. However, the metabolomic profiles of yak colostrum (YC), buffalo colostrum (BC), and bovine colostrum (CC) remain largely unexplored. The objective of this study is to identify unique metabolites that may impact the nutritional value of colostrum. Methods: This study employed ultra-high performance liquid chromatography-electrospray ionization tandem mass spectrometry (UHPLC-ESI-MS/MS) for untargeted metabolomics analysis of YC, BC, and CC. Results: The analysis revealed 97, 70, and 75 differentially expressed metabolites in the YC-CC, BC-CC, and YC-BC comparisons, respectively. In comparison to CC, both YC and BC shared common features, including reduced choline levels and elevated O-acetylcarnitine. Moreover, metabolites such as 2-hydroxy-6-pentadecylbenzoic acid, DL-glycerol-1-phosphate, thiamine, L-carnitine, methyl β-D-galactoside, and uridine diphosphate (UDP) were identified as potential biomarkers for YC, while 21-deoxycortisol, D-synephrine, uridine, mannitol-1-phosphate, nonadecanoic acid, and perillic acid were specific to BC. Conclusions: YC has greater advantages in energy supply, antioxidant activity, immune regulation, and cell homeostasis, and BC holds unique significance in physical development and energy balance regulation. These findings provide valuable insights, enabling the selection of unique bioactive metabolites to develop targeted functional foods from colostrum, catering to diverse nutritional needs.

## 1. Introduction

As a key dietary nutrient, milk offers a complex and well-balanced nutritional profile, mainly including casein and whey protein, which not only supply essential amino acids but also contribute to various physiological functions such as immune modulation and antioxidant activity [1]. Lactose, the main carbohydrate in milk, is essential for regulating intestinal flora and enhancing calcium absorption [1]. With growing consumer demand for health and nutrition, the dairy industry continues to innovate, introducing new products tailored to diverse population needs. Mammalian colostrum, produced within the first three days post-parturition, differs significantly from mature milk in its composition. Its protein content can reach up to 15%, with notable increases in globulin, albumin, and casein levels. Furthermore, colostrum is rich in bioactive compounds such as immunoglobulins, lactoferrin, and growth factors [2], which are vital for immunological defense, growth, and the development of newborns. Research indicates that colostrum can prevent or treat a range of pediatric conditions, including gastrointestinal and respiratory infections, necrotizing enterocolitis, fetal infections, neonatal sepsis, and short-bowel syndrome, among others [3]. Owing to its exceptional nutritional value and health benefits, colostrum holds considerable potential as a functional food resource. The Institute of Food Science and Technology in the United States has identified colostrum as one of the most promising non-herbal natural health foods [4].

The nutritional composition of milk is influenced by various factors, including the type of cattle and their growing environments [5]. Milk from different cattle species often varies significantly in composition. Yaks, for example, are primarily found in cold, high-altitude regions between 3000 and 5000 m above sea level, where they have developed remarkable resilience to cold and low oxygen levels. These extreme conditions have shaped their unique physiological adaptations, which are reflected in the composition of their milk. Referred to as “natural concentrated milk”, yak milk contains significantly higher levels of protein, fat, lactose, and dry matter compared to other animal milk [6]. It has increasingly become a premium dietary resource suitable for newborns, the elderly, and other specific groups. In contrast, buffaloes typically inhabit warm, humid environments such as forests and swamps. As the second-largest global source of milk, buffalo milk offers superior levels of fat, protein, lactose, minerals, and vitamins relative to cow milk. Moreover, it is rich in trace elements like zinc, iron, and calcium, making it particularly beneficial for children’s growth and elderly health needs [7]. Research has demonstrated significant differences in the protein [8] and fatty acids [9] composition between yak colostrum (YC) and mature yak milk, as well as between buffalo colostrum (BC) and mature buffalo milk. However, the metabolic differences between YC, BC, and cow colostrum (CC) remain unexplored.

Metabolomics, an emerging field of biological research, provides powerful tools for analyzing the comprehensive changes in metabolites within complex biological systems. This technology not only allows for quantitative analysis of known metabolites in milk but also facilitates the discovery of new compounds, enabling a more comprehensive understanding of milk’s complexity and diversity and promoting the development of functional foods. For example, Feng et al. [1] employed ultra-high performance liquid chromatography-mass spectrometry (UHPLC-MS/MS) to identify differential metabolites between high- and low-fat milk and analyze the regulatory mechanisms involved. Windarsih et al. [10] utilized ultra-high performance liquid chromatography-high resolution mass spectrometry (UHPLC-HRMS) to investigate the metabolite differences between cow milk, horse milk, and adulterated horse milk, providing a novel approach to detecting adulteration. Similarly, Liu et al. [11] used ^1^H nuclear magnetic resonance (^1^H NMR) metabolomics to determine the effects of drinking cranberry juice or apple juice rich in proanthocyanidins and sugar on human metabolism. These studies illustrate the significant advancements in applying metabolomics to milk composition analysis.

In this study, untargeted metabolomics was employed to map the metabolite profiles of YC, BC, and CC. The differences and metabolic pathways of YC-CC, BC-CC, and YC-BC were systematically compared, and biomarkers for YC and BC were identified. The goal is to clarify the distinct attributes of YC, BC, and CC and to reveal the potential health effects of different animal colostrums, laying a foundation for the development of targeted dairy products in the future.

## 2. Materials and Methods

### 2.1. Chemicals and Reagents

All solvents used in this study were of liquid chromatography-mass spectrometry grade. Methanol and acetonitrile were sourced from Fisher Scientific (Loughborough, UK), ammonium formate from Sigma-Aldrich (Darmstadt, Germany), formic acid from TCI (Shanghai, China), and methyl tert-butyl ether from Sinopharm (Beijing, China). Ultrapure water was produced using a Milli-Q system (Millipore, Bedford, MA, USA).

### 2.2. Collection of Colostrum Samples

The study selected healthy animals, and all colostrum samples were collected within the first 24–48 h postpartum. For each species, a total of 30 samples were randomly collected and then randomly divided into 6 groups (5 samples were pooled for each group). YC samples were collected from yaks raised by individual farmers in Gannan Tibetan Autonomous Prefecture, Gansu Province. The selection criteria for yaks were 5–6 years of age, bearing calves, weighing 210 ± 20 kg, naturally grazed and feeding on the same pasture (the grassland is the subalpine meadows, and the vegetation is dominated by *Cyperaceae* and *Gramineae*), and being in good health. BC samples were provided by Nili-Ravi buffalo from the Chinese Academy of Agricultural Sciences and the Buffalo Research Institute of Guangxi Zhuang Autonomous Region, with inclusion criteria of 5–6 years old, parity of 2–4, weighing 520 ± 20 kg, with consistent diet (alfalfa as the main feed) and environment, and being in good health. Holstein CC samples were collected from cows raised in Daxing, Beijing, selected based on the criteria of 3–7 years old, parity of 2–3, weighing 700 ± 50 kg, with consistent diet (alfalfa as the main feed) and living environment, and in good health. Each animal provided 50 mL of colostrum, manually collected before feeding. Samples were then immediately transported under cold-chain conditions at −20 °C and stored at −80 °C.

### 2.3. Pretreatment of Colostrum Samples

The metabolite extraction procedure followed established protocols [12]. Colostrum samples were removed from −80 °C storage 12 h prior to pretreatment, thawed at 4 °C, and mixed for 1 min. A 20 μL aliquot of each sample was combined with 400 μL of extraction solution (methanol and methyl tert-butyl ether: 1:1, *v*/*v*), mixed for 1 min, and centrifuged at 12,000 rpm at 4 °C for 15 min. The supernatant was filtered through a 0.22 μm membrane, and the resulting filtrate was transferred to a vial for analysis. Equal portions of all sample filtrates were combined to create quality control (QC) samples for machine testing.

### 2.4. UHPLC-ESI-MS/MS

Since colostrum contains a large number of polar, thermally unstable, and macromolecular metabolites, liquid chromatography-mass spectrometry (LC-MS) was used to evaluate the metabolic profiles of YC, BC, and CC to comprehensively identify and quantify a variety of metabolites in dairy products [1]. Ultra-high performance liquid chromatography-electrospray ionization tandem mass spectrometry (UHPLC-ESI-MS/MS) was conducted using a Vanquish UHPLC system (Thermo Fisher Scientific, Waltham, MA, USA) paired with an Orbitrap Exploris 120 mass spectrometer (Thermo Fisher Scientific, Waltham, MA, USA). Each sample (2 μL, six biological replicates for each group) was injected at a flow rate of 0.3 mL/min into a 2.1 × 100 mm, 1.8 μm C18 column (Waters, Milford, MA, USA) maintained at 40 °C. For UHPLC-ESI(+)-MS/MS, the mobile phases were 0.1% (*v*/*v*) formic acid in acetonitrile (B1) and 0.1% (*v*/*v*) formic acid in water (A1). The gradient elution program was as follows: 0–1 min, 8% B1; 1–8 min, 8–98% B1; 8–10 min, 98% B1; 10–10.1 min, 98–8% B1; 10.1–12 min, 8% B1. For UHPLC-ESI(−)-MS/MS, acetonitrile (B2) and ammonium formate (5 mM, A2) served as mobile phases, with the following gradient: 0–1 min, 8% B2; 1–8 min, 8–98% B2; 8–10 min, 98% B2; 10–10.1 min, 98–8% B2; 10.1–12 min, 8% B2 [13]. The mass spectrometry data acquisition used the information-dependent acquisition (IDA) model. MS1 and MS/MS data were acquired simultaneously, with an MS1 resolution of 60,000 FWHM, scanning across an *m*/*z* range of 100–1000. MS/MS resolution was set at 15,000 FWHM. The capillary temperature was maintained at 325 °C, with spray voltages of 3.50 kV for ESI(+) and −2.50 kV for ESI(−). Sheath gas and auxiliary gas pressures were set at 40 and 10 Arb, respectively [14].

### 2.5. Data Processing and Statistical Analyses

The raw mass spectrometry data were converted to mzXML format using Proteowizard-MSConvert (v3.0.8789). Peak detection, filtering, and alignment were performed using R XCMS (v3.12.0) [15]. Systematic errors were identified and corrected through analysis of QC samples, after which metabolites with a coefficient of variance (CV) below 30% were selected for further analysis. Ion identification was based on retention time (RT) and *m*/*z*, and metabolites were cross-referenced with the Human Metabolome Database (HMDB) (http://www.hmdb.ca, accessed on 5 August 2024) [16], MassBank (http://www.massbank.jp/, accessed on 6 August 2024) [17], LipidMaps (http://www.lipidmaps.org, accessed on 5 August 2024) [18], and mzCloud (https://www.mzcloud.org, accessed on 7 August 2024) [19]. Following data normalization, principal component analysis (PCA) and orthogonal partial least squares discriminant analysis (OPLS-DA) were applied to identify outliers and assess deviation trends. Differentially expressed metabolites (DEMs) were then screened based on the OPLS-DA first principal component variable importance for the projection (VIP), along with Student t-tests or analysis of variance (ANOVA) and fold-change (FC) values. After ranking the key DEMs, metabolites with higher levels (VIP > 1, *p* value < 0.05, FC > 4) than those in the other two groups were considered markers. Receiver operating characteristic (ROC) curves were generated for the biomarkers, and the area under the curve (AUC) was calculated. Finally, MetaboAnalyst (http://www.metaboanalyst.ca, accessed on 10 August 2024) was utilized to conduct the Kyoto Encyclopedia of Genes and Genomes (KEGG) pathway enrichment analysis to further investigate the biological significance of the DEMs.

## 3. Results and Discussion

### 3.1. Identification and Multivariate Statistical Comparison of Metabolites Among YC, BC, and CC

**Figure 1:**
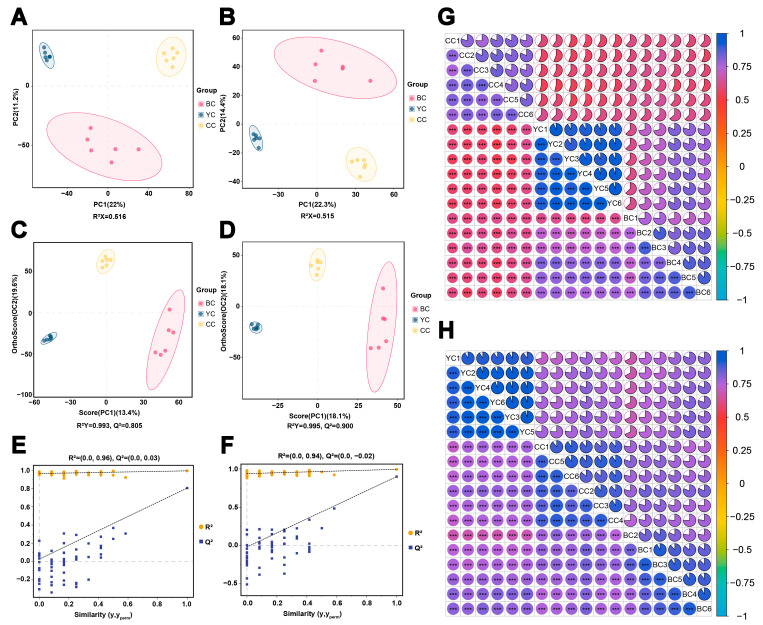
Multivariate analysis of yak colostrum (YC), buffalo colostrum (BC), and cow colostrum (CC). (**A**,**B**) Principal component analysis (PCA) score plots in positive and negative ion modes, respectively. (**C**,**D**) Orthogonal partial least squares discriminant analysis (OPLS-DA) score plots in positive and negative ion modes, respectively. (**E**,**F**) OPLS-DA permutation test plots in positive and negative ion modes, respectively. (**G**,**H**) Correlation heatmaps of samples in positive and negative ion modes, respectively. *** *p* < 0.001.

To investigate the metabolite differences among YC, BC, and CC, UHPLC-ESI-MS/MS was employed in both positive and negative ion modes for data acquisition. The baseline stability of the total ion chromatogram (TIC) (Figure S1A,B) confirmed stable instrument performance, allowing the data to be considered reliable for further analysis. QC and quality assurance (QA) checks, performed using QC samples, indicated a high degree of overlap in both ionization modes (Figure S1C–F), demonstrating the model’s stability and reproducibility across all samples. Multivariate statistical analysis using PCA and OPLS-DA was conducted to examine inter- and intra-group differences. The PCA parameters indicated adequate model fit, with R^2^X values of 0.516 (>0.5) for the positive ion mode and 0.515 (>0.5) for the negative ion mode, suggesting a significant classification of metabolites between YC, BC, and CC. Clear bioreplicate clustering was observed within each group, with some separation attributable to individual variability (Figure 1A,B). In the OPLS-DA model, the positive ion mode yielded model parameters of R^2^Y = 0.993 and Q^2^ = 0.805, while the negative ion mode showed R^2^Y = 0.995 and Q^2^ = 0.900 (Figure 1C,D). To prevent overfitting, 100 permutation tests were conducted (Figure 1E,F), confirming the robustness and predictive capability of the OPLS-DA model. Additionally, the R^2^ and Q^2^ values for comparisons between YC and CC, BC and CC, and YC and BC are presented in Table 1. The VIP values obtained from the OPLS-DA model served as a basis for further analysis of DEMs. The OPLS-DA score plots revealed no overlap among YC, BC, and CC, indicating significant clustering and suggesting distinct compositional differences between the groups, consistent with the PCA results. Comparative analysis of group correlations showed that YC and BC exhibited greater similarity in the positive ion mode (Figure 1G), while BC and CC were more similar in the negative ion mode (Figure 1H). These results suggest notable differences in milk composition among the three groups, and the differential metabolites responsible for sample clustering warrant further exploration.

### 3.2. Metabolic Profile Difference Between YC and CC

**Figure 2:**
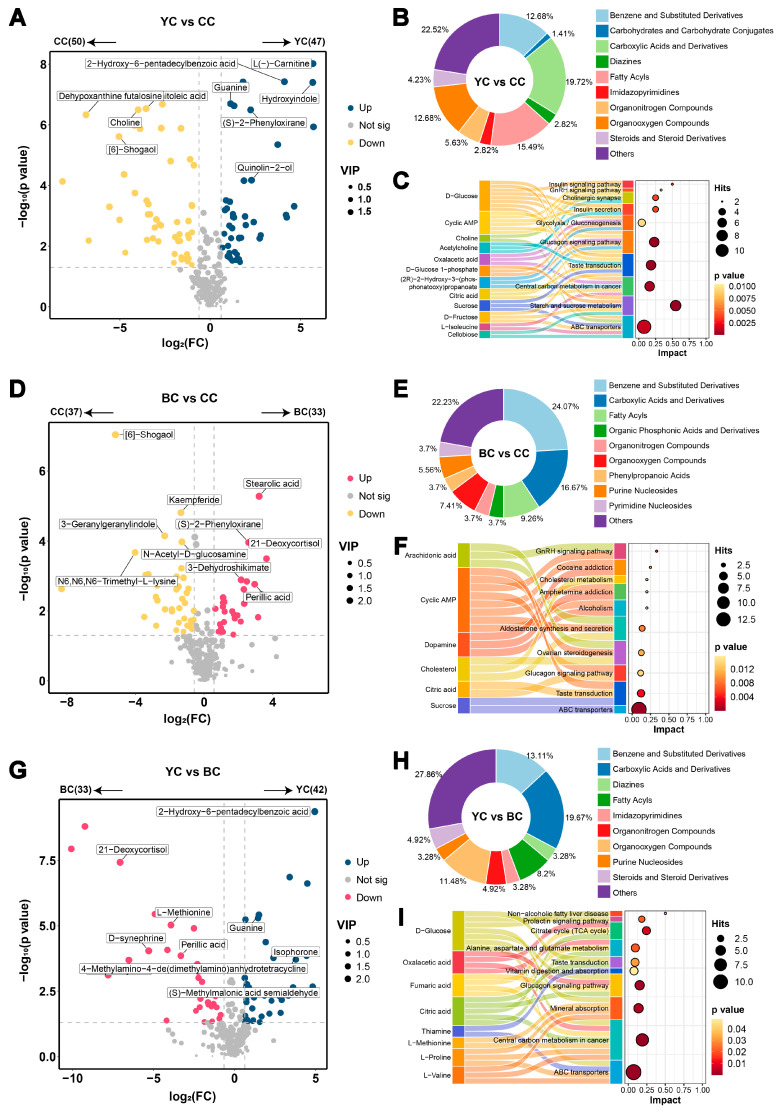
Pairwise analysis of yak colostrum (YC), buffalo colostrum (BC), and cow colostrum (CC). (**A**) Volcano plot of differentially expressed metabolites (DEMs) between YC and CC. (**B**) Subclass composition of DEMs between YC and CC. (**C**) KEGG enrichment analysis presented as a mulberry map and bubble map for DEMs between YC and CC. (**D**) Volcano plot of DEMs between BC and CC. (**E**) Subclass composition of DEMs between BC and CC. (**F**) KEGG enrichment analysis as a mulberry map and bubble map for DEMs between BC and CC. (**G**) Volcano plot of DEMs between YC and BC. (**H**) Subclass composition of DEMs between YC and BC. (**I**) KEGG enrichment analysis in mulberry and bubble maps for DEMs between YC and BC.

To explore the differences in metabolites between YC and CC, 97 DEMs were selected (VIP > 1, *p* value < 0.05, FC > 1.5 or FC < 0.67) and their distribution was visualized using volcanic maps. Compared to CC, YC exhibited significant upregulation in 47 DEMs and downregulation in 50 DEMs (Figure 2A and Table S1). These DEMs, classified by molecular structure, were primarily carboxylic acids and derivatives (19.72%), fatty acyls (15.49%), benzene and substituted derivatives (12.68%), organooxygen compounds (12.68%), organonitrogen compounds (5.63%), steroids and steroids derivatives (4.23%), diazines (2.82%), imidazopyrimidines (2.82%), and carbohydrates and carbohydrate conjugates (1.41%) (Figure 2B). To investigate the mechanisms underlying these metabolic differences, the DEMs were subjected to KEGG enrichment analysis, which identified 10 key metabolic pathways, including starch and sucrose metabolism, the glucagon signaling pathway, taste transduction, central carbon metabolism in cancer, ABC transporters, cholinergic synapse, insulin secretion, insulin signaling pathway, glycolysis/gluconeogenesis, and the GnRH signaling pathway (Figure 2C). Notably, the levels of L-carnitine and O-acetylcarnitine were significantly higher in YC than in CC. Carnitine, especially L-carnitine, is essential in fatty acid oxidation, facilitating the transport of fatty acids into mitochondria for β-oxidation and adenosine triphosphate (ATP) production, which is essential for energy metabolism. Supplementing L-carnitine may enhance energy metabolism and fatty acid utilization [20]. Differences in the levels of D-glucose, sucrose, D-fructose, D-glucose-1-phosphate, cellobiose, sucrose-6-phosphate, and α-maltose-1-phosphate were linked to the enriched starch and sucrose metabolism pathway. This may be attributed to several factors: (1) Yaks require increased energy in high-altitude environments to maintain physiological functions and production, which likely enhances glycogen synthesis through metabolic pathways. (2) Yaks’ unique digestive system, including microbial fermentation in the rumen, enables them to efficiently convert cellulose into energy, producing intermediates related to sucrose and starch metabolism [21]. (3) Yak milk contains relatively higher levels of protein, lactose, and fat compared to cow milk [22], with sucrose and starch metabolites potentially contributing directly to the synthesis of these components in mammary cells. Furthermore, the tricarboxylic acid (TCA) cycle, a key process in cellular respiration and energy production, appeared more active in YC [21], as indicated by lower oxaloacetic acid and higher citric acid levels. Thiamine, which was more abundant in YC, also plays a role in the TCA cycle as a coenzyme for decarboxylase activity. Non-protein nitrogen, an essential component of milk, supports gastrointestinal barrier function and immunity [23]. Additionally, pyrimidine and guanine contents were higher in YC than in CC. Notably, lower levels of choline and acetylcholine were observed in YC, offering valuable insights for designing and producing infant food formulations. This finding could have implications for optimizing nutritional compositions based on specific developmental needs.

### 3.3. Metabolic Profile Difference Between BC and CC

A total of 70 DEMs were identified between BC and CC (VIP > 1, *p* value < 0.05, FC > 1.5 or FC < 0.67), including stearolic acid, 21-deoxycortisol, 6-shogaol, and n6-trimethyl-L-lysine. Of these, 33 metabolites were significantly upregulated while 37 were downregulated in BC (Figure 2D and Table S2). The DEMs primarily comprised benzoic acids and derivatives, amino acids and analogs, and fatty acids and conjugates. Structurally, they were classified as follows: benzene and substituted derivatives (24.07%), carboxylic acids and derivatives (16.67%), fatty acyls (9.26%), organooxygen compounds (7.41%), purine nucleosides (5.56%), organonitrogen compounds (3.7%), phenylpropanoic acids (3.7%), pyrimidine nucleosides (3.7%), and organic phosphonic acids and derivatives (3.7%) (Figure 2E). Further KEGG enrichment analysis revealed key metabolic pathways distinguishing BC from CC, including ABC transporters, taste transduction, GnRH signaling pathway, aldosterone synthesis and secretion, cocaine addiction, ovarian steroidogenesis, glucagon signaling pathway, cholesterol metabolism, amphetamine addiction, and alcoholism (Figure 2F). The analysis highlighted a lower relative concentration of choline in BC compared to CC, consistent with Yang et al. [5], who reported higher choline levels in Chinese Holstein milk. Choline, a key biomarker of mammary immune response, has been linked to mastitis [24]. Both BC and YC showed significantly lower choline levels compared to CC. In contrast, O-acetylcarnitine levels were considerably higher in BC and YC than in CC. In mammary cells, carnitine and acetyl-CoA are esterified to form O-acetylcarnitine, which promotes the entry of fatty acids into mitochondria for β-oxidation and provides energy for the body [25]. Research has demonstrated that cortisol ingested through milk can be absorbed by an infant’s gut [26]. Cortisol facilitates the release of amino acids, glucose, and fatty acids, providing energy for the body, and plays a pivotal role in the development of the hypothalamic–pituitary–adrenal (HPA) axis, influencing stress responses in infants [27]. As a precursor to cortisol, 21-deoxycortisol is essential for cortisol synthesis. Our findings revealed that the concentration of 21-deoxycortisol in BC was approximately 90.86 times higher than in CC, suggesting a potential benefit to infant growth and development. Additionally, the levels of aspartame, sucrose, D-xylose, and citric acid were elevated in BC compared to CC, while the concentrations of L-gulose, D-glucuronic acid, cyclic AMP, and cellobiose were lower. These differences may be attributed to BC’s distinctive anabolic pathways, nutritional composition, and flavor characteristics. Previous studies have shown that buffalo milk contains higher protein levels and is rich in amino acids such as glycine, tyrosine, and cysteine [28]. Concretely, our results indicated increased levels of phenylacetylglycine, γ-glutamylcysteine, and desaminotyrosine in BC, metabolites closely associated with neurotransmitter synthesis, antioxidant functions, and detoxification. Differences in nucleotide metabolism between BC and CC were also observed, with BC showing higher levels of uridine, 9-riburonosyladenine, 1-methyladenosine, and guanosine. Nucleotides, as fundamental components of nucleic acids, are critical for cell proliferation and differentiation. Moreover, nucleotides play a vital role in supporting immune cell development, enhancing intestinal flora composition, and promoting intestinal cell maturation [29]. In summary, the most notable differences between BC and CC were found in the concentrations of choline, 21-deoxycortisol, carbohydrates, amino acids, and nucleotides. These distinctions highlight the unique nutritional and biochemical profiles of BC compared to CC.

### 3.4. Metabolic Profile Difference Between YC and BC

The results indicated that both YC and BC possess high nutritional value. Despite their general similarity, certain significant differences between YC and BC warrant attention. To further explore these differences, a comparative analysis identified 75 DEMs (VIP > 1, *p* value < 0.05, FC > 1.5 or FC < 0.67) between YC and BC. Among these, 42 metabolites, including 2-hydroxy-6-pentadecylbenzoic acid, isophorone, guanine, and S-methylmalonic acid semialdehyde, were more abundant in YC, while 33 metabolites, such as 21-deoxycortisol, L-methionine, perillic acid, and D-synephrine, were present in higher concentrations in BC (Figure 2G and Table S3). These DEMs could be classified as carboxylic acids and derivatives (19.67%), fatty acyls (8.2%), benzene and substituted derivatives (13.11%), organooxygen compounds (11.48%), organonitrogen compounds (4.92%), steroids and steroids derivatives (4.92%), diazines (3.28%), imidazopyrimidines (3.28%), and purine nucleosides (3.28%) (Figure 2H). Mapping these metabolites to the KEGG database revealed 10 significantly enriched metabolic pathways, including central carbon metabolism in cancer, ABC transporters, glucagon signaling pathway, mineral absorption, citrate cycle, alanine, aspartate and glutamate metabolism, prolactin signaling pathway, taste transduction, non-alcoholic fatty liver disease, and vitamin digestion and absorption (Figure 2I). Notable differences between YC and BC were observed in the content of amino acids, vitamins, and fatty acids. In terms of amino acids, YC showed higher levels of L-valine and L-proline, while BC was richer in L-methionine. L-valine, an essential branched-chain amino acid, is essential for muscle health and promotes protein synthesis and repair, serving as a source of acetyl-CoA [5]. On the other hand, L-methionine, another essential amino acid that must be obtained through diet, is abundant in BC and supports infant development, cell health, and immune function [30]. Vitamin content also varied significantly between YC and BC. YC contained 3.58 times more pyridoxal phosphate and 7.51 times more thiamine than BC, whereas BC had significantly higher retinol levels, with YC containing only 0.05 times that of BC. These variations in nutritional composition suggest opportunities for developing functional foods with specific health benefits. For instance, YC could be used in products designed to promote nervous system development and immunity, while BC could support functional foods aimed at improving vision and enhancing bone growth [31]. In terms of fatty acids, BC exhibited higher levels of linoleic acid and stearolic acid, likely influenced by the animals’ diet [32]. Optimizing feed formulations during animal rearing could potentially increase the levels of these fatty acids in YC.

### 3.5. Characteristic Metabolic Biomarkers of YC

**Figure 3:**
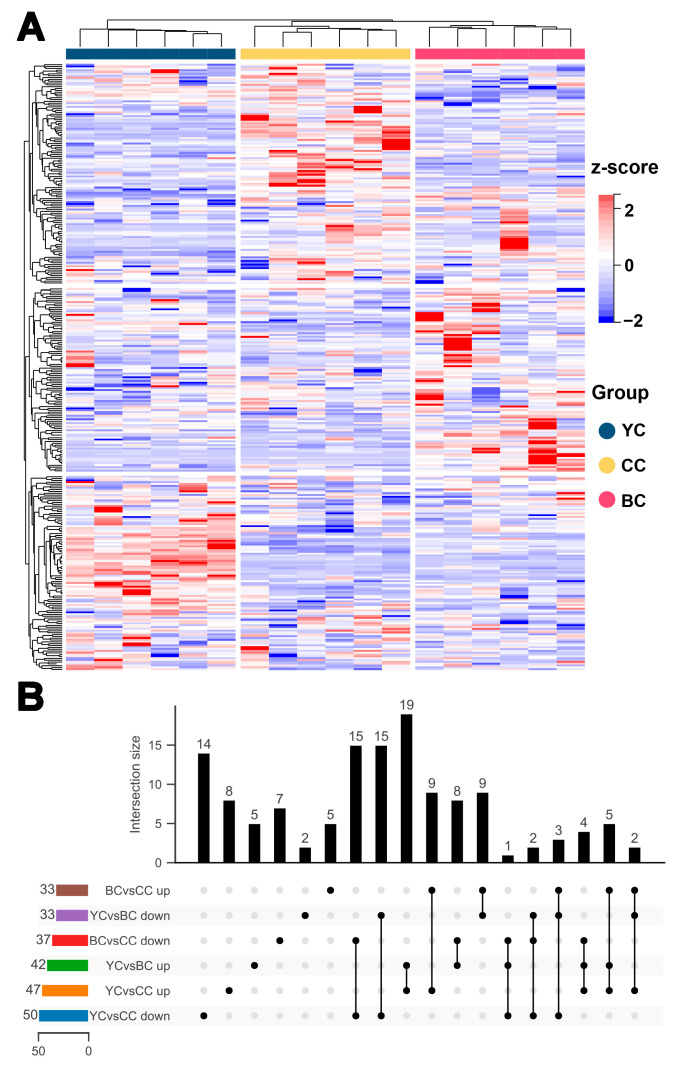
Clustering and distribution of all differentially expressed metabolites (DEMs) from pairwise analysis of yak colostrum (YC), buffalo colostrum (BC), and cow colostrum (CC). (**A**) Heatmap showing all DEMs across YC, BC, and CC. (**B**) Upset plot displaying the overlap and unique DEMs among YC, BC, and CC.

**Figure 4:**
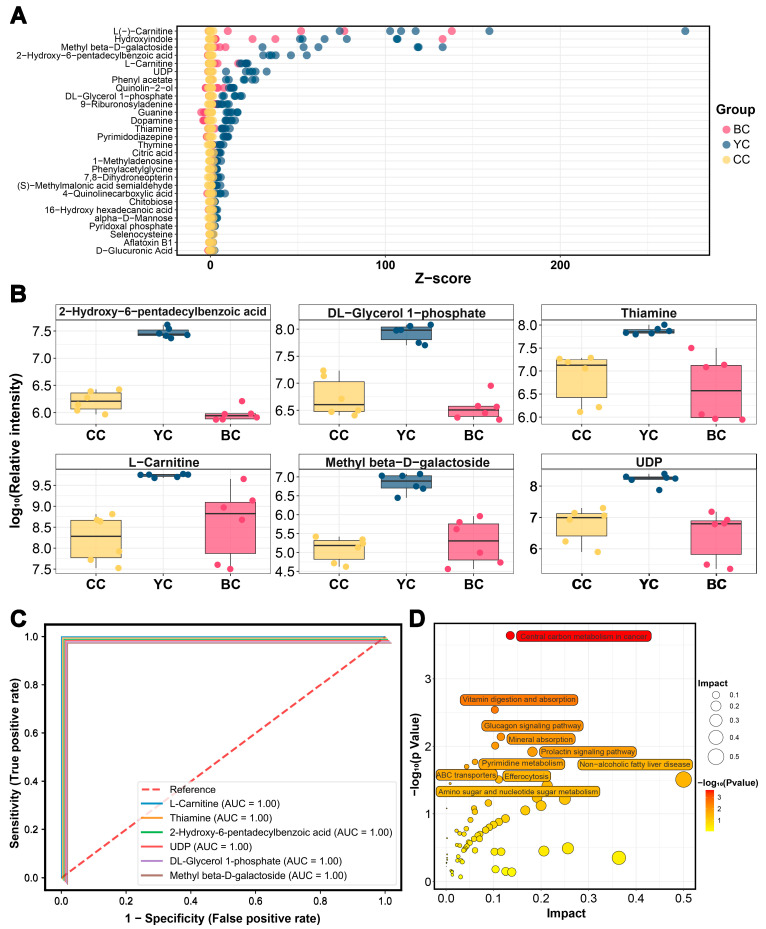
Metabolite markers of yak colostrum (YC). (**A**) Z-score map of differentially expressed metabolites (DEMs) with higher expression in YC compared to BC and CC. (**B**) Relative content of 2-hydroxy-6-pentadecylbenzoic acid, DL-glycerol-1-phosphate, thiamine, L-carnitine, methyl β-D-galactoside, and uridine diphosphate (UDP) across YC, BC, and CC. (**C**) Receiver operating characteristic (ROC) curves for 2-hydroxy-6-pentadecylbenzoic acid, DL-glycerol-1-phosphate, thiamine, L-carnitine, methyl β-D-galactoside, and UDP. (**D**) KEGG pathway enrichment analysis of DEMs with differential expression in YC compared to BC and CC.

To further investigate the differences among YC, BC, and CC, a comparative analysis of the clusters of DEMs identified in pairwise analyses was conducted, revealing 133 DEMs across the three groups (VIP > 1, *p* value < 0.05, FC > 1.5 or FC < 0.67) (Figure 3A). Specifically, 28 DEMs were significantly higher in YC compared to BC and CC, while 20 were lower (Figure 3B and Figure 4A). The KEGG functional enrichment analysis is shown in Figure 4D. To identify potential metabolite biomarkers that differentiate YC from BC and CC, DEMs with FC > 4 were further screened, and ROC curves were plotted to assess their specificity, including 2-hydroxy-6-pentadecylbenzoic acid (AUC = 1.00), DL-glycerol-1-phosphate (AUC = 1.00), thiamine (AUC = 1.00), L-carnitine (AUC = 1.00), methyl β-D-galactoside (AUC = 1.00), and uridine diphosphate (UDP) (AUC = 1.00) (Figure 4B,C). 2-hydroxy-6-pentadecylbenzoic acid (anacardic acid), a histone acetyltransferase (HAT) inhibitor, plays a significant role in gene regulation [33]. It has been reported to possess antibacterial [34], antioxidant [35], and anti-inflammatory [36] properties. Our study revealed that 2-hydroxy-6-pentadecylbenzoic acid levels in YC were 17 times higher than in CC and 30 times higher than in BC. DL-glycerol-1-phosphate, a key precursor for phospholipid synthesis, participates in cell membrane formation and indirectly influences cell signaling. It is essential for maintaining cell structure and function [37]. In YC, DL-glycerol-1-phosphate was found to be 11 times more abundant than in CC and 22 times higher than in BC, suggesting that YC may have a stronger role in promoting nervous system development and maintaining cell membrane integrity. The results also indicated that YC contained significantly higher levels of thiamine, approximately 6–7 times that of CC or BC. Thiamine, an essential vitamin, is involved in numerous enzymatic reactions. This suggests that YC could serve as a valuable nutritional supplement to support thiamine intake. The L-carnitine content in YC was approximately 18 times higher than in CC and 4 times higher than in BC, facilitating lipid metabolism and providing antioxidant benefits. Li et al. [38] reported that yak milk contains roughly twice the fat content of cow milk, likely contributing to the elevated L-carnitine levels in YC. Methyl β-D-galactoside, linked to β-D-galactose, was found to be 52 and 21 times higher in YC compared to CC and BC, respectively. As a key component of the oligosaccharide backbone, β-D-galactoside supports energy provision and intestinal health [39]. Additionally, UDP was 18 and 28 times more abundant in YC than in CC and BC. Alongside lactose and oligosaccharides, mammalian milk contains glyconucleotides such as UDP-Glc, UDP-Gal, and UDP-GlcNAc, which are essential for the synthesis of UDP-sugars involved in regulating growth and immune function in newborns [40]. Overall, YC offers superior advantages in energy provision, antioxidant capacity, and cell homeostasis due to its distinct environmental and dietary factors, making it a valuable resource for producing high-quality, nutrient-rich foods.

### 3.6. Characteristic Metabolic Biomarkers of BC

**Figure 5:**
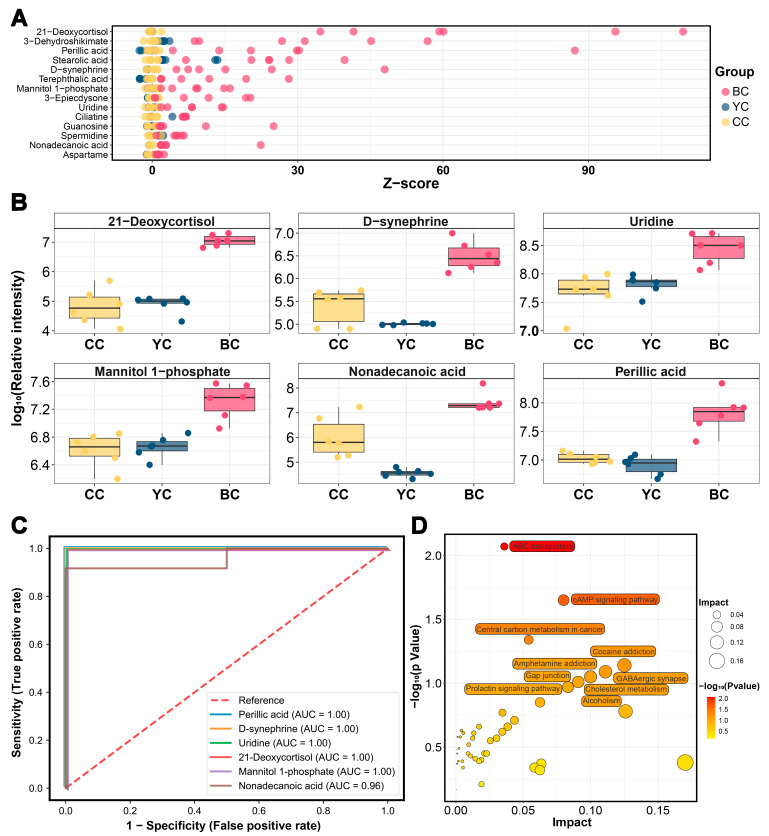
Metabolite markers of buffalo colostrum (BC). (**A**) Z-score map of differentially expressed metabolites (DEMs) with higher expression in BC compared to YC and CC. (**B**) Relative content of 21-deoxycortisol, D-synephrine, uridine, mannitol-1-phosphate, nonadecanoic acid, and perillic acid across YC, BC, and CC. (**C**) The receiver operating characteristic (ROC) curves for 21-deoxycortisol, D-synephrine, uridine, mannitol-1-phosphate, nonadecanoic acid, and perillic acid. (**D**) KEGG pathway enrichment analysis of DEMs with differential expression in BC compared to YC and CC.

Similarly, levels of 14 DEMs were significantly higher in BC compared to YC and CC, while 13 were lower (VIP > 1, *p* value < 0.05, FC > 1.5 or FC < 0.67) (Figure 3B and Figure 5A). The corresponding KEGG enrichment is shown in Figure 5D. Further screening of metabolite biomarkers for BC, combined with ROC curve analysis, identified 21-deoxycortisol (AUC = 1.00), D-synephrine (AUC = 1.00), uridine (AUC = 1.00), mannitol-1-phosphate (AUC = 1.00), nonadecanoic acid (AUC = 0.96), and perillic acid (AUC = 1.00) as potential biomarkers for BC (Figure 5B,C). D-synephrine, an alkaloid, has been shown to reduce oxidative stress by inhibiting NF-κB and MAPK signaling pathways and mitigating diabetic symptoms [41]. Bai et al. [42] further demonstrated that synephrine regulates energy balance by influencing amino acid metabolism in mice on a high-fat diet. The concentration of D-synephrine in BC was 12 times higher than in CC and 39 times higher than in YC. Mannitol-1-phosphate, a key intermediate in the mannitol metabolic pathway, was found to be five times more abundant in BC than in CC or YC. Mannitol, widely used in the food and pharmaceutical industries, is a low-metabolic, non-glycemic sweetener with osmotic pressure regulation and antioxidant properties [43]. Additionally, BC exhibited higher levels of perillic acid, with concentrations 7 and 10 times greater than those in CC and YC, respectively. As a primary metabolite of D-limonene, perillic acid has radiation protection effects and promotes bone marrow and α-esterase positive cell production [44,45]. Uridine, a critical RNA component, is involved in genetic information transmission, cell proliferation, and differentiation, as well as the regulation of energy intake and metabolic balance [46]. Uridine levels in BC were five times higher than in CC and YC. Nonadecanoic acid and 21-deoxycortisol were also elevated in BC, though their specific biological functions remain unclear and require further investigation. Overall, BC demonstrates distinct biological significance and potential applications in immune regulation and energy metabolism.

## 4. Conclusions

This study analyzed the metabolic differences among YC, BC, and CC to uncover the distinct nutrients in colostrum from different breeds. UHPLC-ESI-MS/MS results identified 97 DEMs between YC and CC, including L-carnitine, O-acetylcarnitine, and metabolites related to starch and sucrose metabolism as well as the TCA cycle. Between BC and CC, 70 DEMs were identified, such as 21-deoxycortisol, aspartame, sucrose, D-xylose, citric acid, uridine, 9-riburonosyladenine, 1-methyladenosine, and guanosine. Additionally, 75 DEMs distinguished YC from BC, including L-valine, L-proline, L-methionine, pyridoxal phosphate, thiamine, and retinol. These findings highlight significant metabolic discrepancies among the three groups, warranting further exploration. Both YC and BC exhibited lower choline and higher O-acetylcarnitine levels compared to CC, and CC had no significant advantages compared to others. After further analysis, key biomarkers for YC were identified, including 2-hydroxy-6-pentadecylbenzoic acid, DL-glycerol-1-phosphate, thiamine, L-carnitine, methyl β-D-galactoside, and UDP. For BC, 21-deoxycortisol, D-synephrine, uridine, mannitol-1-phosphate, nonadecanoic acid, and perillic acid emerged as potential biomarkers. These results suggested that YC has greater advantages in energy supply, antioxidant activity, immune regulation, and cell homeostasis and is suitable for developing products that promote nervous system development and immunity, while BC holds unique significance in physical development and energy balance regulation to support functional foods that improve vision, promote bone growth, and control blood sugar levels. The limitations of this study were the relatively small sample size and the exclusion of biologically active compounds such as proteins. Future research will further expand the sample size and examine the main components of colostrum from proteins, especially the IgG content in terms of metabolism and the formation process. In summary, this study offers valuable insights for the development of novel dairy products tailored to meet the specific nutritional needs of various populations.

## Figures and Tables

**Table 1:** Statistical information of the orthogonal partial least squares discriminant analysis (OPLS-DA) score models in positive and negative ion modes, respectively.

Comparison	R^2^X		R^2^Y		Q^2^	
	Positive Mode	Negative Mode	Positive Mode	Negative Mode	Positive Mode	Negative Mode
YC vs. CC	0.389	0.413	1.000	1.000	0.950	0.949
BC vs. CC	0.334	0.364	1.000	0.997	0.903	0.880
YC vs. BC	0.342	0.427	0.999	0.999	0.902	0.960

Abbreviations are YC, yak colostrum; BC, buffalo colostrum; CC, cow colostrum; R^2^X, goodness of fit (R^2^) X cumulative; R^2^Y, goodness of fit (R^2^) Y cumulative; Q^2^, goodness of prediction.

## Data Availability

The original contributions presented in this study are included in the article/Supplementary Material. Further inquiries can be directed to the corresponding author.

## Supplementary Materials

The following supporting information can be downloaded at: https://www.mdpi.com/article/10.3390/foods14020232/s1, Figure S1: Data check of yak colostrum (YC), buffalo colostrum (BC), and cow colostrum (CC); Table S1: The differential metabolites identified between yak colostrum (YC) and cow colostrum (CC) (VIP > 1, *p* value < 0.05, FC > 1.5 or FC < 0.67); Table S2: The differential metabolites identified between buffalo colostrum (BC) and cow colostrum (CC) (VIP > 1, *p* value < 0.05, FC > 1.5 or FC < 0.67); Table S3: The differential metabolites identified between yak colostrum (YC) and buffalo colostrum (BC) (VIP > 1, *p* value < 0.05, FC > 1.5 or FC < 0.67).

## Abbreviations

The following abbreviations are used in this manuscript:

YC	yak colostrum
BC	buffalo colostrum
CC	cow colostrum
UHPLC-MS/MS	ultra-high-performance liquid chromatogre-mass spectrometry
UHPLC-HRMS	ultra-high-performance liquid chromatogre-high resolution mass spectrometry
1H NMR	1H nuclear magnetic resonance
QC	quality control
LC-MS	liquid chromatography-mass spectrometry
UHPLC-ESI-MS/MS	ultra-high-performance liquid chromatography-electrospray ionization tandem mass spectrometry
CV	coefficient of variance
RT	retention time
HMDB	Human Metabolome Database
PCA	principal component analysis
OPLS-DA	orthogonal partial least squares discriminant analysis
DEMs	differential expression metabolites
VIP	variable importance for the projection
FC	fold-change
ROC	receiver operating characteristic
AUC	area under the curve
KEGG	Kyoto Encyclopedia of Genes and Genomes
TIC	total ion chromatogram
QA	quality assurance
R2X	goodness of fit (R2) X cumulative
R2Y	goodness of fit (R2) Y cumulative
Q2	goodness of prediction
TCA	tricarboxylic acid
ATP	adenosine triphosphate
HPA	hypothalamic–pituitary–adrenal
HAT	histone acetyltransferases
UDP	uridine diphosphate
