# Supplementary material for: Untargeted Metabolomics Reveals Key Differences Between Yak, Buffalo, and Cow Colostrum Based on UHPLC-ESI-MS/MS
Source: Foods. 2025 Jan 13;14(2):232. doi: 10.3390/foods14020232 (PMC11765268; doi:10.3390/foods14020232)
Supplement: Supplementary file 1 [file foods-14-00232-s001.zip › foods-3417864-supplementary.pdf]

Figure S1. Data check of yak colostrum (YC), buffalo colostrum (BC) and cow colostrum (CC). (A) and (B) Plots of total ion chromatogram (TIC) in positive and negative ion mode, respectively. (C) and (D) Principal component analysis (PCA) score plots of quality control (QC) in positive and negative ion mode, respectively. (E) and (F) PCA score plots of quality assurance (QA) in positive and negative ion mode, respectively.

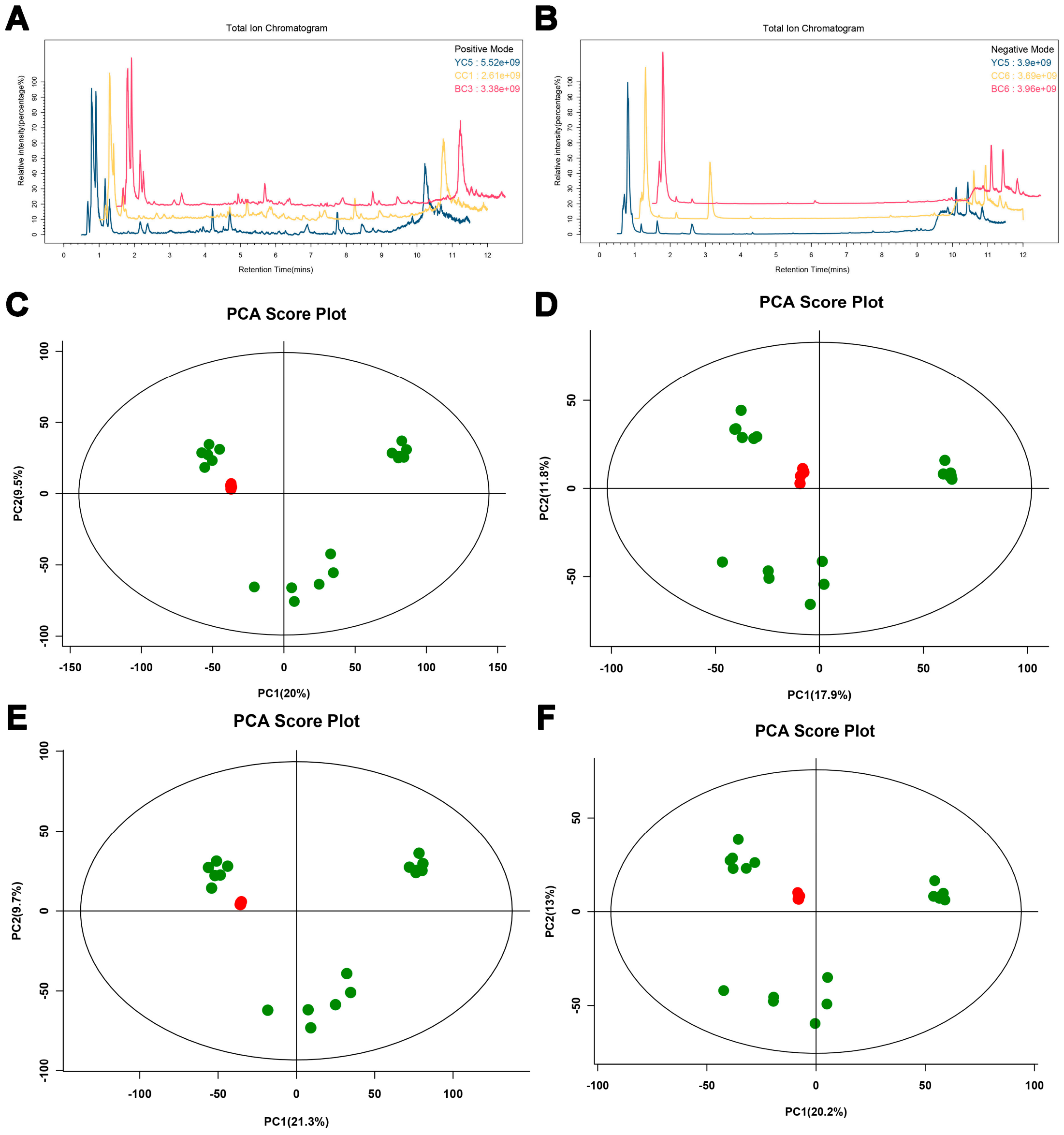

Table S1. The differential metabolites identified between yak colostrum (YC) and cow colostrum (CC) (VIP > 1， p value < 0.05, FC > 1.5 or FC < 0.67).

Metabolite	FC	p value	VIP
(S)-Methylmalonic acid semialdehyde	1.76	0.001	1.486
Epsilon-caprolactam	1.89	0.001	1.486
(S)-2-Phenyloxirane	4.73	0.000	1.705
Thymine	2.13	0.000	1.522
Hydroxyindole	51.15	0.000	1.726
Phosphonoacetate	1.94	0.022	1.255
Quinolin-2-ol	4.88	0.000	1.699
Guanine	2.19	0.000	1.712
L-Carnitine	18.89	0.001	1.597
L(-)-Carnitine	51.58	0.000	1.736
Selenocysteine	2.57	0.016	1.284
O-Acetylcarnitine	2.47	0.001	1.574
Pyrimidodiazepine	5.31	0.002	1.561
N6-Acetyl-LL-2,6-diaminoheptanedioate	2.14	0.025	1.250
Confertifolin	3.04	0.006	1.314
7,8-Dihydroneopterin	2.08	0.002	1.400
Stearolic acid	3.03	0.005	1.318
Thiamine	6.69	0.006	1.448
9-Riburonosyladenine	3.66	0.000	1.589
1-Methyladenosine	3.81	0.001	1.544
Aflatoxin B1	2.69	0.028	1.220
Sucrose	2.35	0.030	1.101
2-Hydroxy-6-pentadecylbenzoic acid	17.31	0.000	1.726
Uridine diphosphate	18.41	0.001	1.564

Metabolite	FC	p value	VIP
Chitobiose	3.14	0.033	1.177
Caproic acid	1.84	0.000	1.484
1,2,3-Trihydroxybenzene	1.72	0.004	1.308
L-Ribulose	1.61	0.011	1.285
Dopamine	2.49	0.000	1.672
Phenyl acetate	13.36	0.000	1.627
Spermidine	1.99	0.010	1.350
DL-Glycerol-1-phosphate	11.99	0.000	1.588
α-D-Mannose	2.23	0.005	1.273
Uric acid	25.1	0.000	1.595
4-Quinolinecarboxylic acid	3.02	0.027	1.088
D-Fructose	2.39	0.001	1.421
(2R)-2-Hydroxy-3-(phosphonatooxy)propanoate	3.53	0.000	1.686
Citric acid	6.97	0.004	1.420
Phenylacetylglycine	3.28	0.005	1.405
D-Glucuronic acid	2.08	0.017	1.170
Methyl β-D-galactoside	52.76	0.000	1.652
Pyridoxal phosphate	2.78	0.019	1.150
γ-Glutamylcysteine	2.42	0.001	1.505
D-Glucose-1-phosphate	2.29	0.029	1.078
Dehydroepiandrosterone	2.06	0.021	1.125
16-Hydroxy hexadecanoic acid	2.32	0.003	1.461
α-Maltose-1-phosphate	3.92	0.002	1.496
Methylmalonic acid	0.38	0.026	1.120
Choline	0.06	0.000	1.705
Aminohydroquinone	0.13	0.001	1.566
Maltol	0.29	0.007	1.402

Metabolite	FC	p value	VIP
Creatine	0.49	0.000	1.503
cis-4-Hydroxy-D-proline	0.14	0.002	1.545
p-Aminobenzoic acid	0.5	0.000	1.503
Acetylcholine	0.53	0.000	1.623
L-Methionine	0.07	0.000	1.687
Diphenylamine	0.31	0.004	1.343
L-Theanine	0.15	0.000	1.687
N6,N6,N6-Trimethyl-L-lysine	0.1	0.001	1.459
Glycylleucine	0.33	0.023	1.144
Kynurenic acid	0.11	0.006	1.410
N-Acetyl-D-glucosamine	0.33	0.000	1.689
Propionylcarnitine	0.33	0.028	1.115
Palmitoleic acid	0.09	0.000	1.707
Retinol	0.02	0.001	1.596
6-Shogaol	0.03	0.000	1.737
Oleic acid	0.41	0.018	1.248
Dehypoxanthine futalosine	0.01	0.000	1.702
12-Keto-tetrahydro-leukotriene B4	0.09	0.000	1.511
3-Geranylgeranylindole	0.22	0.000	1.635
4-Methylamino-4-de(dimethylamino)anhydrotetracycline	0.03	0.001	1.544
3-Epiecdysone	0.12	0.002	1.383
Oxalacetic acid	0.46	0.000	1.599
Benzoate	0.26	0.018	1.256
L-Isoleucine	0.15	0.002	1.500
L-Xylonate	0.04	0.000	1.662
3-(2-Hydroxyphenyl)propanoic acid	0.09	0.000	1.486
D-Ornithine hydrochloride	0.25	0.035	1.130

Metabolite	FC	p value	VIP
D-Glucose	0.31	0.007	1.267
Terephthalic acid	0.28	0.002	1.394
Salicyluric acid	0.44	0.001	1.495
D-Tryptophan	0.04	0.016	1.166
Galactaric acid	0.44	0.005	1.286
Myristoleic acid	0.09	0.001	1.427
Epiandrosterone	0.05	0.000	1.651
Linoleic acid	0.42	0.032	1.058
(6Z)-Octadecenoic acid	0.5	0.003	1.316
Stearic acid	0.09	0.006	1.378
Nonadecanoic acid	0.01	0.007	1.394
Arachidonic acid	0.06	0.000	1.599
trans-Cinnamoyl-β-D-glucoside	0.26	0.005	1.413
N-Glycolylneuraminic acid	0.16	0.000	1.670
Cyclic AMP	0.49	0.041	1.035
Cellobiose	0.43	0.004	1.310
Chenodeoxycholic acid	0	0.000	1.543
Lincomycin	0.14	0.007	1.396
Sucrose-6-phosphate	0.44	0.025	1.093

Table S2. The differential metabolites identified between buffalo colostrum (BC) and cow colostrum (CC) (VIP > 1， p value < 0.05, FC > 1.5 or FC < 0.67).

Metabolite	FC	p value	VIP
Epsilon-caprolactam	1.58	0.009	1.524
(S)-2-Phenyloxirane	6.05	0.000	1.886
Hydroxyindole	23.05	0.003	1.634
Phosphonoacetate	1.91	0.024	1.451
Perillic acid	7.75	0.002	1.838
D-synephrine	12.44	0.000	1.827
3-Dehydroshikimate	4.46	0.001	1.869
O-Acetylcarnitine	2.27	0.006	1.573
N6-Acetyl-LL-2,6-diaminoheptanedioate	3.37	0.013	1.463
Confertifolin	4.39	0.020	1.398
Uridine	5.62	0.001	1.719
Stearolic acid	9.23	0.000	1.997
Aspartame	2	0.040	1.375
9-Riburonosyladenine	2.16	0.004	1.747
1-Methyladenosine	2.14	0.008	1.539
Sucrose	2.23	0.021	1.393
21-Deoxycortisol	90.86	0.000	1.996
Cholesterol	2.16	0.006	1.561
3-Epiecdysone	11	0.004	1.620
Ciliatine	5.05	0.006	1.714
Spermidine	3.61	0.000	1.773
D-Xylose	3.11	0.010	1.486
Terephthalic acid	4.98	0.002	1.687
Desaminotyrosine	2.16	0.040	1.283
Phthalic acid	2.3	0.010	1.500

Metabolite	FC	p value	VIP
(2R)-2-Hydroxy-3-(phosphonatooxy)propanoate	3.27	0.048	1.304
Citric acid	3.46	0.016	1.529
Phenylacetylglycine	1.98	0.031	1.294
Methyldopa	1.79	0.039	1.282
Mannitol-1-phosphate	5.14	0.001	1.794
γ-Glutamylcysteine	8.9	0.015	1.596
Guanosine	3.87	0.013	1.465
Nonadecanoic acid	10.16	0.003	1.609
Choline	0.62	0.033	1.396
L-Valine	0.33	0.001	1.735
Aminohydroquinone	0.27	0.010	1.635
cis-4-Hydroxy-D-proline	0.36	0.022	1.382
p-Aminobenzoic acid	0.5	0.003	1.644
Isophorone	0.1	0.001	1.756
7-Methylguanine	0.33	0.002	1.676
Diphenylamine	0.45	0.016	1.421
N6,N6,N6-Trimethyl-L-lysine	0.06	0.000	1.853
N-Acetyl-D-glucosamine	0.41	0.000	1.884
Pyrimidodiazepine	0.32	0.035	1.291
Procainamide	0.09	0.015	1.592
Cytidine	0.51	0.026	1.423
Palmitoleic acid	0.37	0.008	1.520
6-Shogaol	0.03	0.000	2.062
Oleamide	0.42	0.004	1.611
Dehypoxanthine futalosine	0.14	0.026	1.474
Bisoprolol	0.59	0.019	1.408
2-Hydroxy-6-pentadecylbenzoic acid	0.56	0.028	1.329
Oxybutynin	0.15	0.005	1.719

Metabolite	FC	p value	VIP
3-Geranylgeranylindole	0.2	0.000	1.910
Succinic acid semialdehyde	0.66	0.018	1.417
Benzoate	0.21	0.009	1.658
Dopamine	0.66	0.010	1.529
Aminosalicylic acid	0.62	0.021	1.356
Hippuric acid	0.43	0.001	1.833
L-Gulose	0.44	0.039	1.227
D-Glucuronic acid	0.15	0.002	1.657
D-Tryptophan	0	0.002	1.829
Myristoleic acid	0.09	0.001	1.724
Inosine	0.46	0.006	1.598
Kaempferide	0.39	0.000	1.960
Arachidonic acid	0.14	0.004	1.604
Cyclic AMP	0.27	0.009	1.507
Cellobiose	0.4	0.001	1.757
Lincomycin	0.36	0.002	1.824
Glycocholic acid	0.53	0.002	1.633

Table S3. The differential metabolites identified between yak colostrum (YC) and buffalo colostrum (BC) (VIP > 1，p value < 0.05, FC > 1.5 or FC < 0.67).

Metabolite	FC	p value	VIP
(S)-Methylmalonic acid semialdehyde	1.55	0.001	1.895
L-Proline	1.66	0.046	1.394
L-Valine	2	0.004	1.798
Thymine	1.68	0.004	1.777
Hydroxyindole	2.22	0.041	1.405
Isophorone	10.64	0.000	1.989
Quinolin-2-ol	3.18	0.029	1.471
Guanine	2.84	0.000	2.003
Glucosamine	1.64	0.001	1.818
L-Carnitine	4.45	0.023	1.518
L(-)-Carnitine	2.92	0.048	1.334
7-Methylguanine	5.28	0.000	1.884
Selenocysteine	2.17	0.044	1.347
Pyrimidodiazepine	16.35	0.003	1.821
Procainamide	7.16	0.021	1.541
7,8-Dihydroneopterin	1.66	0.008	1.557
Thiamine	7.51	0.007	1.754
9-Riburonosyladenine	1.69	0.002	1.713
1-Methyladenosine	1.78	0.002	1.674
Oleamide	2.13	0.014	1.592
Aflatoxin B1	10	0.005	1.781
Chlorpromazine	1.59	0.028	1.416
2-Hydroxy-6-pentadecylbenzoic acid	30.89	0.000	2.114
Oxybutynin	6.93	0.005	1.760
Lacto-N-biose I	1.71	0.038	1.380

Metabolite	FC	p value	VIP
Uridine diphosphate	28.29	0.002	1.842
Chitobiose	1.53	0.045	1.260
Fumaric acid	1.78	0.044	1.194
Dopamine	3.78	0.000	1.804
Phenyl acetate	10.54	0.000	1.837
DL-Glycerol-1-phosphate	22.49	0.000	1.826
α-D-Mannose	1.85	0.002	1.482
4-Quinolinecarboxylic acid	4.85	0.007	1.381
Hippuric acid	1.59	0.000	1.777
Citric acid	2.02	0.004	1.430
Phenylacetylglycine	1.65	0.007	1.394
D-Glucuronic acid	13.78	0.000	1.735
Methyl β-D-galactoside	21.69	0.000	1.669
D-Tryptophan	12.25	0.002	1.601
Pyridoxal phosphate	3.58	0.005	1.430
16-Hydroxy hexadecanoic acid	2.3	0.006	1.542
Kaempferide	2.72	0.000	1.783
L-Methionine	0.07	0.000	1.986
Perillic acid	0.1	0.000	1.890
D-synephrine	0.03	0.000	2.058
3-Dehydroshikimate	0.28	0.003	1.830
L-Theanine	0.28	0.048	1.324
Glycylleucine	0.37	0.012	1.592
Vanillylmandelic acid	0.52	0.035	1.413
Porphobilinogen	0.41	0.010	1.522
Uridine	0.21	0.001	1.793
Palmitoleic acid	0.23	0.006	1.589
Stearolic acid	0.33	0.004	1.632

Metabolite	FC	p value	VIP
Retinol	0.05	0.042	1.364
Aspartame	0.32	0.008	1.661
21-Deoxycortisol	0.01	0.000	2.092
4-Methylamino-4-de(dimethylamino)anhydrotetracycline	0	0.001	1.933
3-Epiecdysone	0.01	0.000	1.984
Oxalacetic acid	0.41	0.004	1.547
Ciliatine	0.25	0.001	1.692
Spermidine	0.55	0.025	1.325
L-Xylonate	0.04	0.000	1.792
3-(2-Hydroxyphenyl)propanoic acid	0.19	0.018	1.247
D-Glucose	0.46	0.013	1.387
Terephthalic acid	0.06	0.000	1.689
Galactaric acid	0.44	0.005	1.419
Mannitol-1-phosphate	0.2	0.000	1.632
Epiandrosterone	0.03	0.000	1.788
Linoleic acid	0.32	0.010	1.335
Guanosine	0.23	0.013	1.410
Nonadecanoic acid	0	0.000	1.860
trans-Cinnamoyl-β-D-glucoside	0.21	0.001	1.571
N-Glycolylneuraminic acid	0.18	0.000	1.754
Chenodeoxycholic acid	0	0.000	1.869
Lincomycin	0.38	0.045	1.176
